# A Scoping Review of Evidence-Based Grief Interventions for Parentally Bereaved Children: Comparing Individual, Group, and Family Approaches

**DOI:** 10.1177/00302228251334249

**Published:** 2025-04-14

**Authors:** Marcus Gottlieb, Dominic Violo, Jacqueline Tams, Jamie Piercy

**Affiliations:** Department of Psychology, 97950 University of British Columbia Okanagan, Kelowna, BC, Canada

**Keywords:** grief interventions, parentally bereaved children, bereavement, objective outcomes, therapeutic formats, scoping review methodology, evidence-based interventions

## Abstract

This scoping review examines and compares the effectiveness of different intervention formats for parentally bereaved children by focusing on three therapeutic formats: individual, group, and family-based approaches. Using the PRISMA-ScR guidelines, we analyzed ten peer-reviewed quantitative studies published between 2010 and 2022. This study emphasizes formal interventions with measurable outcomes, excluding creative or symbolic approaches such as art and ritual-based therapies. This review highlights that individual interventions, particularly cognitive-behavioural techniques, significantly reduce critical deficits such as prolonged grief disorder and PTSD symptoms. Group approaches foster social connection and emotional regulation, while family interventions emphasize improved family communication and caregiver support. Notable limitations include small sample sizes, heterogeneity in program structure, and reliance on self-reported outcomes. Future research should explore diverse grief experiences, longer-term impacts, and cultural considerations. This review provides insights into tailoring interventions to the unique needs of bereaved children, aiding practitioners in selecting evidence-based approaches.

## Introduction

A Scoping Review of Evidence-Based Grief Interventions for Parentally Bereaved Children: Comparing Individual, Group, and Family Approaches

In developed nations, it has historically been estimated that about 3–4% of young people lose a parent before reaching adulthood (Lutzke et al., 1997). However, more recent evidence suggests that this number may be closer to 5% (Chater et al., 2022) or even 7% (Burns et al., 2020). Regardless of prevalence rates, the death of a parent is a tragic event for young people, often leading to a cascade of difficulties. Indeed, early parental loss is associated with a range of adverse outcomes, including pathological levels of anxiety and depression (Breier et al., 1988), as well as prolonged grief reactions—defined as grief persisting beyond normal expectations and interfering with daily functioning (Alvis et al., 2023). Additionally, children who lose a parent during childhood are at an increased risk of diminished self-esteem (Angelhoff et al., 2021), decreased happiness (Moor & de Graaf, 2016), increased risk for suicide (Guldin et al., 2015), substance abuse difficulties (Hamdan et al., 2019), and eating problems (Johnson et al., 2002). Challenges with executive functioning (Slomine & Copeland-Linder, 2021), which are likely linked to deficits in academic functioning (Liu et al., 2022), have also been observed.

Although some of these adverse outcomes appear to remit over time, many of these challenges persist into adulthood, including socioeconomic disadvantages, workplace challenges, and increased familial conflict (Feigelman et al., 2018). These difficulties are often, at least in part, attributable to the complex and enduring grief responses that follow the loss of a parent. Happiness levels in parentally bereaved individuals also appear to be diminished compared to their non-bereaved peers for at least ten years after a parent’s death (Moor & de Graaf, 2016).

Children’s grief responses to losing a parent are heterogeneous. Several internal and external factors surrounding a parent’s death influence the manifestation and extent of a child’s grief response. These factors include but are not limited to, the suddenness of the death, the deceased parent’s relationship with the child, the availability of supports and other attachment figures (e.g., the surviving parent, siblings, grandparents), peer relationships, the child’s age, and their personal resilience (Dopp & Cain, 2012; Howell et al., 2015; Kailaheimo-Lönnqvist & Erola, 2020; Melhem et al., 2011). However, regardless of these circumstances, both qualitative and quantitative evidence suggests that the challenges associated with losing a parent can be at least partially mitigated through formal interventions (Bergman et al., 2017; Chater et al., 2022; Neimeyer & Currier, 2009; Rosner et al., 2010).

This review focuses on assessing formal interventions for parentally bereaved children, defined as structured support programs or therapies designed to help children cope with the emotional and psychological impact of losing a parent (Currier et al., 2007). We examined professionally delivered interventions with measurable outcomes, excluding creative, symbolic, or informal support approaches (e.g., art or ritual-based therapies), to maintain methodological consistency. By including only studies with objectively measurable outcomes, we are ensuring reliable comparisons of quantitative intervention effectiveness.

These interventions address both preventive support, aimed at mitigating risk factors for future psychological disorders, and treatment support for children with existing clinical concerns. Put simply, such interventions aim to provide emotional support, develop coping strategies, and mitigate the adverse effects associated with parental loss, such as anxiety, depression, and prolonged grief. Interventions can be delivered at the family, individual, and/or group levels, all with strengths, weaknesses, and various levels of apparent efficacy. They will now be considered sequentially.

### Individual Interventions

Individual interventions for grieving children aim to provide emotional support, normalize children’s experiences after the loss of a parent, and offer a safe environment for expressing emotions and thoughts (Kentor & Kaplow, 2020). Ultimately, these interventions focus on facilitating the child’s grieving process and improving both physical and mental health after loss (Bergman et al., 2017). Core components of such interventions typically include grief psychoeducation, emotion identification and regulation skills, cognitive coping and restructuring, grief and trauma processing, memorializing and maintaining bonds, meaning-making, and future planning (Kentor & Kaplow, 2020). Furthermore, these interventions are designed to enhance children’s self-esteem, adaptive control beliefs, coping skills, and healthy emotional expression while fostering a positive parent-child relationship with the surviving parent (Haine et al., 2008).

Research has demonstrated that individual interventions can be effective in reducing traumatic grief—grief compounded by exposure to events involving sudden, violent, or otherwise highly distressing circumstances surrounding a parent’s death (Pynoos & Nader, 1990)—and posttraumatic distress symptoms in children (Currier et al., 2007). Specifically, evidence suggests that trauma-informed cognitive behavioural therapy (TF-CBT) and Trauma and Grief Component Therapy for Adolescents (TGCT-A) tend to be beneficial for children whose grief is complicated by trauma (Thompson et al., 2019). These approaches help children process their grief and trauma, leading to improvements in emotional regulation and mental health outcomes (Currier et al., 2007; Thompson et al., 2019). Moreover, interventions incorporating components included in these approaches, such as cognitive restructuring and emotion regulation, have been shown to help children better cope with their loss and improve their overall well-being (Cohen et al., 2016). Scholars and practitioners alike emphasize the necessity of offering individually tailored support to parentally bereaved children, considering the diverse reactions children may have to losing a parent (Hill et al., 2019; Kentor & Kaplow, 2020).

Despite the apparent effectiveness of individual interventions, they also have several limitations. One major limitation is the lack of universal guidelines for these interventions, which can lead to variability in their implementation and outcomes (Kentor & Kaplow, 2020). Additionally, many studies on the efficacy of individual interventions are limited by small sample sizes and short durations, making it challenging to assess long-term effects (Bergman et al., 2017; Currier et al., 2007; Rosner et al., 2010). Furthermore, individual interventions may not fully address the diverse needs of all grieving children, necessitating the inclusion of other supports, such as family or group interventions (Haine et al., 2008). Finally, the effectiveness of these interventions can be constrained by the subjective nature of self-reported outcomes and the need for culturally sensitive approaches tailored to the child’s developmental stage and cultural background (Bergman et al., 2017; Thompson et al., 2019).

### Group Interventions

Group interventions for parentally bereaved children, while sharing goals with familial and individual interventions, are unique in providing a supportive environment for children to express their feelings and share experiences with peers who have undergone similar losses (Huss & Ritchie, 1999). These interventions often include structured activities such as camps or retreats (e.g., Fluegeman et al., 2013; McClatchey & Wimmer, 2014) to help children process grief, develop coping skills, and, crucially, reduce feelings of isolation. Activities may include creating memory books, discussing feelings, and learning about grief stages. The primary goals of group interventions are to improve emotional well-being, enhance self-concept, reduce symptoms of depression and anxiety, increase awareness about grief, reduce isolation, provide a space for sharing feelings, and teach coping strategies (O'Brien et al., 2022). Additionally, they aim to offer a sense of normalcy and community through shared experiences (Abuhegazy & Elkeshishi, 2017).

The effectiveness of group interventions appears to vary. Studies have shown that well-structured group therapy can significantly reduce symptoms of depression and anxiety and improve self-concept among bereaved children. For instance, Abuhegazy and Elkeshishi (2017) found their 12-week school-based supportive therapy group for bereaved children demonstrated significant improvement in depression, anxiety, and self-concept. Similarly, after analyzing 17 studies, Bergman et al. (2017) highlighted that structured group programs can lead to reductions in traumatic grief symptoms and improvements in social adjustment and peer relationships. Congruently, an evaluation of a school-based grief group for 14 elementary school students reported increased well-being and healthy coping skills (Tillman & Prazak, 2018). A case study of grieving children participating in a school-based grief intervention also found positive outcomes (i.e., reduced isolation and increased ability to discuss feelings; Ross & Hayes, 2004). Lastly, in England, a qualitative investigation of 23 bereaved children who attended grief groups also found reduced isolation among the participants (Metel & Barnes, 2011).

However, other studies, such as one conducted by Huss and Ritchie (1999), reported no significant changes in self-esteem, depression, or behaviour following a group intervention. These findings were attributed to small sample sizes and short intervention durations (Abuhegazy & Elkeshishi, 2017; Bergman et al., 2017). Perhaps most concerning, a meta-analysis of 13 quantitative studies found little to no support for the overall effectiveness of grief interventions for children at the group level (Currier et al., 2007).

Despite the positive outcomes observed in some studies, notable limitations persist in group interventions for parentally bereaved children. One significant limitation is the variability in program duration and structure, which can impact the consistency and generalizability of results (Currier et al., 2007; O'Brien et al., 2022). Additionally, the subjective nature of self-reported outcomes and the lack of long-term follow-up in many studies pose challenges in assessing the enduring effects of these interventions (O'Brien et al., 2022). Moreover, while group settings provide valuable peer support, they may not address an individual child’s specific needs (Bergman et al., 2017).

### Familial Interventions

The surviving caregiver/parent’s reaction to their children’s grief is essential for determining how it manifests and persists (Hung & Rabin, 2009). Indeed, evidence suggests that children who have recently lost a parent typically depend heavily on their surviving parent to facilitate their grief and mourning (Kwok et al., 2005; Shapiro et al., 2014). While many surviving parents play a central role in supporting their children through grief, they may also face challenges in providing adequate support due to the stress of single parenting, managing household responsibilities, and coping with their own grief (Kwok et al., 2005). Family support programs aim to enhance parents’ capacity to provide emotional stability and guidance following their loss. Further, due to changes in the family structure and roles after a death, children may end up supporting their remaining parents while managing their own grief and, often, associated psychological difficulties (Shapiro et al., 2014). These considerations frequently result in children receiving less time, attention, and support from their remaining parent or caregiver than they need, as the surviving parent has to navigate their own grief while transitioning to a new parenting role (Bergman et al., 2017; Shapiro et al., 2014).

Thus, it is logical that bereavement interventions target bereaved family members (namely the remaining parent) as well—not just the children. Indeed, research has shown that most extant interventions designed to support parentally bereaved children also target their remaining caregivers (Bergman et al., 2017). The objectives of these interventions include enhancing family communication and the caregiver-child relationship, aiding the grieving process, improving health, strengthening parenting skills, increasing stability and predictability for the children, and reducing adverse events among them (Kwok et al., 2005). These interventions are typically brief, ranging from six to 14 sessions, and are conducted by professionals such as social workers or counsellors with extensive experience in child guidance, grief, or psychiatry (Bergman et al., 2017; Currier et al., 2007; Rosner et al., 2010).

Bereavement programs, like the Family Bereavement Program (Sandler et al., 2003, 2010), have shown many positive outcomes, including reductions in children’s grief-related distress and improvements in their mental health. These interventions have been effective in enhancing parental warmth and family communication, which are critical factors in helping children adapt to the loss of a parent (Sandler et al., 2010). Studies have reported medium to large effects on reducing traumatic grief symptoms in children and improving the mental health of caregivers. For instance, in their review, Bergman et al. (2017) found that structured family programs can lead to significant improvements in both children’s and caregivers’ well-being, emphasizing the importance of supporting the surviving parents to enable them to help their children better (Giang et al., 2024).

However, limitations to family interventions exist, too. One significant drawback is that similar to group interventions, they often do not sufficiently focus on the children’s individual needs, which can be crucial for their adjustment and recovery (Christ et al., 2005). Unfortunately, children may be overlooked in favour of the surviving parent. Additionally, similar to the other intervention formats, the effectiveness of these interventions can be constrained by variability in program duration and structure, affecting the consistency of results (Bergman et al., 2017; Christ et al., 2005). Moreover, the subjective nature of self-reported outcomes and the lack of long-term follow-up in many studies pose challenges in assessing the enduring effects of these interventions (Bergman et al., 2017; Rosner et al., 2010).

### Present Study

Given the increasing recognition of the diverse needs of parentally bereaved children (e.g., Feigelman et al., 2018), exploring how to maximize the support provided to this population is crucial. Although direct comparisons between these formats are limited at present, understanding which approaches offer the greatest benefits provides valuable insights for best practice recommendations. Moreover, various support programs are available; however, there is a significant gap in research comparing the effectiveness of individual, family, and group intervention formats (Bergman et al., 2017; Rosner et al., 2010).

To address this gap, we are conducting a scoping review, given its unique ability to allow for a comprehensive overview of intervention by mapping the range of evidence available without the constraints of a systematic review’s narrower focus (Arksey & O’Malley, 2005; Munn et al., 2018). While systematic reviews are valuable for synthesizing high-quality evidence on intervention efficacy by rigorously appraising and integrating findings from controlled studies, their stringent inclusion criteria may exclude relevant but heterogeneous research on grief interventions, making a scoping review more suitable for capturing the breadth of available evidence in this field (Arksey & O'Malley, 2005; Munn et al., 2018). Addressing this gap will help family members, treatment providers, and other interested parties identify which intervention formats may offer the most benefit for specific demographics and circumstances, even as further research is needed to develop and validate each type of intervention fully. Therefore, this study aims to assess the relative effectiveness of these three delivery formats for childhood grief interventions.

Specifically, this research will evaluate each format’s strengths, weaknesses, and outcomes by conducting a comprehensive scoping review, following the methodological framework outlined by Arksey and O'Malley (2005) and the PRISMA-ScR guidelines (Tricco et al., 2018). By examining the extant literature on the topic, this scoping review aims to provide a detailed comparison that can inform the development of tailored intervention strategies, ensuring that bereaved children receive the most appropriate and practical support for their unique needs. This investigation will contribute to a deeper understanding of how to optimize psychosocial outcomes for parentally bereaved children by identifying which intervention formats work best in various contexts and for different individuals.

## Method

### Search Strategy

In November 2022, initial literature searches were conducted using Boolean search strings formulated to address the primary research goals. An additional search was conducted in July 2024 to ensure the inclusion of recent studies. As such, the search was based on the delivery format of the intervention (family, group, and individual were all included in search terms; see Table 1), the nature of the intervention, and the population receiving the intervention. Relevant terms related to each category were linked using the Boolean operator ‘OR'; the three categories were then combined using the Boolean operator ‘AND.' Considering that studies examining grief support span multiple academic disciplines, searches were performed in databases that index publications across various fields. These databases included MEDLINE, PsycINFO, Sociological Abstracts, and PubMed. Additionally, hand searching was used to ensure key studies the authors knew should be included in the review were indeed included. The search captured all such studies.

**Table 1.** Search Strings Related to Format, Intervention and Population.

Area	Search string
Delivery format of intervention	“Group therap*” OR “Group counseling” OR “Group intervention*” OR “Group treatment*” “Family therap*” OR “Family counseling” OR “Family intervention*” OR “Family treatment*” OR “Individual therap*” OR “Individual counseling” OR “Individual intervention*” OR “Individual treatment*”
Nature of the intervention	“Grief intervention*” OR “Grief treatment*” OR “Grief therap*” OR “Child bereave*” OR “Child grief” OR “Parental loss”, “Parental bereave*”
Population	Child* or Adolescen* or You* or Teen*

### Selection Process

Articles were selected according to predefined inclusion and exclusion criteria (see Table 2). Our inclusion criteria specifically focused on grief interventions that provided quantitative outcomes for children up to 18 years old who were parentally bereaved. Moreover, we prioritized quantitative studies to enable consistent comparisons of intervention outcomes. In contrast, qualitative studies and reviews were excluded to maintain methodological consistency, ensuring that measurable outcomes could be compared across intervention formats. Studies exclusively focused on medication were also excluded, while studies combining medication with psychosocial interventions were reviewed for relevance but ultimately excluded if medication outcomes dominated.

**Table 2.** Inclusion and Exclusion Criteria.

	Inclusion criteria	Exclusion criteria
Population	• Parentally bereaved children	• Only target of the intervention is someone other than a parentally bereaved child up to 18 years of age
	• Up to 18 years of age • Lost one or both parent	
Study design	• Experimental study	• Literature reviews
	• Pre/post design	• Background articles
		• Qualitative research
		• Case studies
Intervention	• A form of intervention for grief or bereavement following the loss of a parent	• Medication
	• Delivered in individual, group, and/or family format	
Outcomes	• Measurable outcomes reported (i.e., quantitative)	• Non-empirically measurable outcomes reported (e.g., qualitative)
		• No outcomes reported
Publication type	• Peer-reviewed published articles	• Non-peer-reviewed articles (e.g., newspaper articles)
	• Publication between 2000 and 2022	• Master’s theses
	• In English	• Doctoral Dissertations
		• Published in a language other than English

Although we acknowledge that children can experience various types of grief (e.g., disenfranchised grief, collective grief, ambiguous loss) and that grief can arise from different types of loss (e.g., losing a non-related caregiver or sibling), this research was deliberately targeted toward children who had lost one or both parents. This focus was chosen to align with the research objectives and previous literature, allowing for a comprehensive but precise examination of interventions with measurable outcomes for parentally bereaved children.

The initial database searches yielded 983 articles, including 357 duplicates, which resulted in 626 studies being reviewed for title and abstract screening. Two reviewers independently assessed each study based on predefined inclusion and exclusion criteria. Discrepancies were resolved through discussion and consultation with a third reviewer to ensure consistency and reliability in the selection process. At this stage, 579 studies were excluded, primarily due to lack of relevance, inappropriate study design (including insufficient outcome data), or an ineligible population. This left 47 articles eligible for full-text screening, after which an additional set of studies was excluded for similar reasons, with the most common being inappropriate study design, an ineligible population, or lack of relevance. Following this process, ten articles were retained for inclusion in the final review. Refer to the PRISMA-ScR (Tricco et al., 2018) flowchart (Figure 1) for a detailed overview of the screening process steps, which follow the scoping review extension guidelines for systematic reporting.

**Figure 1.**
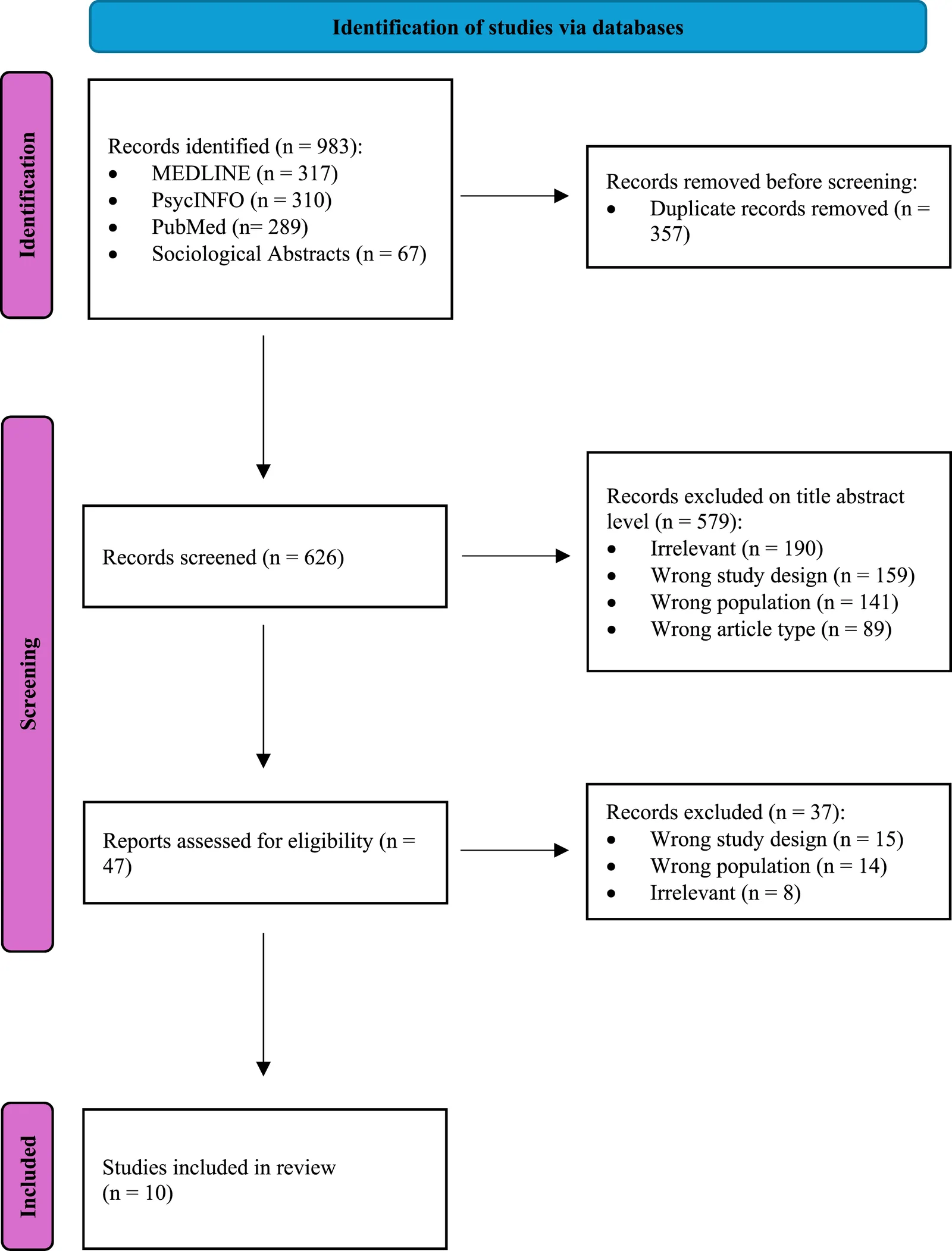
PRISMA flow Chart.

### Data Extraction and Analysis

The data was extracted using a manual that had been developed prior to data extraction, consistent with Peter et al.'s (2021) scoping review guidance. Two reviewers independently extracted data on study identifiers, delivery format, participants, study design, outcomes measured, and key findings. The manual was iteratively refined based on feedback from both reviewers during pilot testing of the first ten studies. Data analysis involved synthesizing descriptive statistics and categorizing outcomes by intervention type. After iteration, the extracted data eventually comprised information about the study’s identifying information (e.g., author, year, journal, etc.), delivery format, study design, participants, outcomes measured, outcomes found, the content of grief support, and the study’s outcome (see Table 3).

**Table 3.** Overview of Included Studies.

In	Author (year)	Participants	Country	Delivery format	Aim of study	Study design	Outcomes measured	Outcomes found
1	Boelen et al. (2021)	134 children and adolescents with prolonged grief	Netherlands	Individual	To examine the effects of a CBT program, CBT grief-help, for prolonged grief disorder in children and adolescents	A randomized clinical trial comparing CBT grief-help to supportive counselling	Prolonged grief disorder, depression, PTSD, and problem behaviour	CBT grief-help resulted in greater reductions in prolonged grief disorder symptoms, depression, PTSD, and internalizing problems compared to supportive counselling
2	De Groot et al. (2010)	122 relatives of suicide victims with high suicide ideation	Netherlands	Family	To examine the effectiveness of family-based cognitive-behavioural grief therapy in preventing complicated grief in relatives of suicide victims	Randomized controlled trial with follow-up at 13 months	Complicated grief, depression, suicide ideation, maladaptive grief reactions	Grief therapy reduced maladaptive grief reactions and suicide ideation among suicide ideators compared to non-ideators
3	Hill et al. (2019)	65 bereaved youth aged 6–17 years	United States	Individual	To evaluate Multidimensional grief therapy for reducing maladaptive grief, PTSD, and depressive symptoms in bereaved youth	Pilot open trial with single-group design	Maladaptive grief reactions, PTSD symptoms, depressive symptoms	Significant reductions in maladaptive grief, PTSD, and depressive symptoms with large effect sizes
4	Kalantari et al. (2012)	61 war-bereaved Afghani refugee adolescents aged 12–18 years	Iran	Individual	To examine the efficacy of ‘writing for recovery' on traumatic grief symptoms among Afghani refugee adolescents	Randomized control trial with experimental and control groups	Traumatic grief symptoms using the traumatic grief inventory for Children (TGIC)	Significant reduction in traumatic grief symptoms in the experimental group compared to the control group
5	Linder et al. (2022)	296 children and youth aged 11–18 years	United States	Group	To evaluate the effectiveness of an 8-week, school-based grief group treatment program	Quasi-experimental pre-post design with no control group	Grief symptomology, emotion dysregulation, social support	Significant reduction in grief symptoms, frequency of emotion dysregulation, and increased perceived social support; no significant change in dysregulation intensity
6	MacKinnon et al. (2015)	26 parentally bereft children actively seeking support	Canada	Group	To assess the feasibility of a Meaning-Based group Counseling (MBGC) intervention with a randomized controlled trial (RCT) design	Pilot RCT with experimental (MBGC) and control (bereavement support group) arms	Meaning in life, anxiety, depression, grief	MBGC was feasible with no substantial attrition; most participants completed all questionnaires and reported acceptable measures
7	McClatchey and Wimmer (2014)	32 bereaved children and adolescents aged 6–18 years	United States	Individual	To examine predictors of posttraumatic growth (PTG) and the impact of trauma-informed care on PTG among bereaved youth	Preexperimental, pretest–posttest design	Posttraumatic growth (PTG)	Participants showed a statistically significant increase in PTG scores after attending the trauma-informed care healing camp
8	Sandler et al. (2010)	244 parentally bereaved youth aged 8–16 years	United States	Family	To assess the long-term effects of the family Bereavement Program (FBP) on multiple indicators of grief	Randomized experimental trial with four assessments over six years	Problematic grief (IGTS), Texas revised inventory of grief (TRIG), inventory of traumatic grief (ITG)	FBP reduced problematic grief (IGTS) at post-test and six-year follow-up and reduced social Detachment/Insecurity at six-year follow-up for low grief levels, older youth, and boys
9	Siddaway et al. (2015)	168 children attending CHUMS bereavement groups, aged 3–16 years	United Kingdom	Group	To evaluate the effectiveness of CHUMS child bereavement service using statistical and clinical significance methodology	Naturalistic one-group pre-post design	Psychological problems using the strengths and difficulties questionnaire (SDQ)	Statistically significant, a medium size decrease in symptoms over time for parents and children, a slight decline for teachers, and 13–18% of participants showed reliable and clinically significant improvement
10	Spuij et al. (2015)	10 bereaved children and adolescents aged 10–18 years	Netherlands	Individual, family	To present outcomes of ‘grief-help,' a cognitive-behavioural treatment for prolonged grief in children and adolescents	Open trial with consecutive cases	Prolonged grief disorder (PGD), depression, PTSD, internalizing and externalizing problems	Significant improvements in self-rated PGD and PTSD, moderate improvements in depression, and small to moderate improvements in internalizing and externalizing problems

## Results

### Overview of Selected Studies

Ten studies were included in this scoping review. All studies had measurable outcomes and focused on interventions that supported children who experienced the death of a parent. The studies were peer-reviewed and published from 2010 to 2022. Each study received an identification number (IN) to simplify the discussion (see Table 3).

The scoping review encompasses ten studies focusing on grief interventions for children and adolescents. The participants included were all parentally bereaved children and adolescents ranging between 3 and 18 years old. The countries represented in these studies include the United States (*n* = 4; 3, 5, 7, 8), the Netherlands (*n* = 3; 1, 2, 10), Iran (*n* = 1; 4), Canada (*n* = 1; 6), and the United Kingdom (*n* = 1; 9). Per our research question, the delivery formats of the interventions vary across individual (*n* = 5), group (*n* = 3), and family (*n* = 3) levels. Namely, individual-level interventions were implemented in studies 1, 3, 4, and 7. Group-level interventions were conducted in studies 5, 6, and 9, while family-level interventions were present in studies 2 and 8. Study 10 was categorized at both the individual and family level.

The aims of the studies ranged from examining the effects of cognitive-behavioral therapies and grief-specific interventions (1, 2, 3, 4, 5, 10) to evaluating predictors of posttraumatic growth (7) and long-term effects of bereavement programs (6, 8, 9). The outcomes measured across these studies included prolonged grief disorder (1, 3, 4, 10), depression (1, 3, 4, 5, 6, 10), PTSD (1, 3, 10), problem behaviour (1, 10), complicated grief (2), suicide ideation (2), maladaptive grief reactions (3, 4), emotion dysregulation (5), social support (5), meaning in life (6), anxiety (6), posttraumatic growth (7), and other psychological concerns (8, 9).

### Individual Level Interventions

The individual delivery format was used in studies 1, 3, 4, 7, and 10. These studies involved clinical trials (1, 4), pilot open trials (3), pre-experimental pre-post designs (7), and open trials with consecutive cases (10). The outcomes showed significant reductions in prolonged grief disorder (1, 3, 4, 10), depression (1, 3, 4, 10), PTSD (1, 3, 10), problem behaviour (1, 10), and maladaptive grief reactions (3, 4). Table 3 includes details for all included studies. However, as an example, Study 1 implemented the CBT Grief-Help intervention, consisting of 12 weekly sessions, each lasting 90 minutes. This intervention included components such as trauma processing, cognitive restructuring, and emotion regulation. Effectiveness was assessed using standardized measures, including the Prolonged Grief Disorder Scale (PGDS) and the Child PTSD Symptom Scale (CPSS), with results showing significant reductions in prolonged grief symptoms, depression, and PTSD compared to supportive counselling (Boelen et al., 2021). Moreover, in terms of outcomes, study 3 found significant decreases in maladaptive grief, PTSD, and depressive symptoms with large effect sizes. In study 4, the ‘Writing for Recovery' intervention showed substantial reductions in traumatic grief symptoms. Study 10 revealed considerable improvements in self-rated prolonged grief disorder and PTSD, moderate improvements in depression, and small to moderate improvements in internalizing and externalizing problems.

### Group Level Interventions

Group-level interventions were evaluated in studies 5, 6, and 9 (see Table 3). Study 5 assessed an 8-week school-based group program designed to improve emotional regulation and peer support. Each session incorporated activities such as guided discussions, role-playing, and expressive art exercises. Effectiveness was assessed using pre- and post-intervention measures, including the Emotion Dysregulation Scale and a peer support inventory, revealing significant reductions in grief symptoms and increased perceived social support. Study 6 assessed the feasibility of a Meaning-Based Group Counseling (MBGC) intervention for parentally bereaved children, measuring outcomes such as meaning in life, anxiety, depression, and grief. The results indicated that the MBGC intervention was feasible, with no substantial attrition, and participants reported acceptable measures across all outcomes. Additionally, study 9 used a naturalistic one-group pre-post design to evaluate the CHUMS child bereavement service, which included children attending bereavement groups aged 3–16 years. The outcomes of study 9 revealed a statistically significant, medium-sized decrease in psychological symptoms for parents and children over time, with a slight reduction for teachers. Moreover, 13–18% of participants showed reliable and clinically significant improvement.

### Family Level Interventions

Family-level interventions were examined in studies 2, 8, and 10. Study 2 utilized a randomized controlled trial with follow-up at 13 months to assess the effectiveness of family-based cognitive-behavioural grief therapy for relatives of suicide victims. The study found that grief therapy reduced maladaptive grief reactions and suicide ideation among suicide ideators compared to non-ideators. Study 8 used the Family Bereavement Program (FBP), a 12-session family-focused intervention targeting both children and their caregivers. Sessions emphasized enhancing family communication, problem-solving skills, and emotional expression. Effectiveness was assessed with four follow-ups over six years, utilizing the Texas Revised Inventory of Grief and the Inventory of Traumatic Grief. The study found that the Family Bereavement Program (FBP) significantly reduced problematic grief at both the post-test and the six-year follow-up, highlighting the potential for long-term benefits when interventions focus on family dynamics and communication. Additionally, it reduced social detachment and insecurity at the six-year follow-up, particularly among participants with mild grief symptoms, older youth, and boys. Another study, which included individual-level interventions (Study 10), demonstrated that the ‘Grief-Help' program led to improvements in self-reported prolonged grief disorder, PTSD, and depression.

### Comparison Overview

When comparing the three intervention formats, it becomes apparent that individual-level interventions showed the most substantial reductions in a wide range of symptoms, including prolonged grief disorder, depression, PTSD, and problem behaviours. This format appears particularly effective for children and adolescents directly experiencing grief-related symptoms. Group-level interventions effectively reduced grief symptoms and improved social support and emotional regulation, suggesting they are beneficial for fostering social connections and emotional processing in a peer setting. Family-level interventions were effective in addressing maladaptive grief reactions and reducing psychological symptoms, particularly for relatives of suicide victims and parentally bereaved youth. These interventions seem most effective when there is a need to address family dynamics and support children and their caregiver(s).

Thus, based on the available evidence, individualized interventions appear particularly effective for addressing specific personal grief symptoms. However, comparable social and emotional support data in individual and family interventions were not collected, limiting direct comparisons. This gap highlights the need for future research to explore these outcomes across all intervention types to strengthen the evidence base for practice recommendations. Therefore, while trends suggest potential efficacy differences across formats, further research is needed to confirm these observations.

## Discussion

Given the significant and widespread adverse effects on children who have lost a parent (e.g., Breier et al., 1988; Feigelman et al., 2018) and the potential for interventions to support these children and mitigate adverse effects (Rosner et al., 2010), it is crucial to determine the most effective way to deliver these interventions to support this vulnerable population. To do so, scholars have called for research into elucidating the differences between individual, group, and family interventions (i.e., delivery format) for such interventions (Bergman et al., 2017; Rosner et al., 2010).

Overall, the results of our scoping review suggest that there is no one clear “winner”; particular circumstances and goals must be considered when determining who should receive which intervention. Specifically, the individual format might be superior for directly targeting personal grief symptoms (Boelen et al., 2021; Hill et al., 2019; Kalantari et al., 2012; Spuij et al., 2015), the group format for social and emotional support (Linder et al., 2022; Siddaway et al., 2015), and the family format for addressing grief within a familial context, especially in cases of suicide and parental loss (de Groot et al., 2010; Sandler et al., 2010; Spuij et al., 2015).

These results are in line with previous literature that has found that complicated grief symptoms can be reduced following individualized cognitive behavioural therapy (e.g., Rosner et al., 2011). Furthermore, evidence suggests that providing individually tailored treatments may be the most crucial factor in reducing the development of complicated grief (Simon, 2013). The studies in our scoping review indicated that individualized grief interventions led to significant reductions in prolonged grief disorder (Boelen et al., 2021; Kalantari et al., 2012; Spuij et al., 2015), depression (Boelen et al., 2021; Hill et al., 2019; Kalantari et al., 2012; Spuij et al., 2015), PTSD (Boelen et al., 2021; Hill et al., 2019; Spuij et al., 2015), and problem behaviour (Boelen et al., 2021; Spuij et al., 2015). For instance, targeted cognitive behavioural therapy, specifically CBT Grief-Help, resulted in more significant reductions in these symptoms than supportive counselling (Boelen et al., 2021). Additionally, the ‘Writing for Recovery' intervention effectively reduced traumatic grief symptoms in war-bereaved adolescents (Kalantari et al., 2012), while Multidimensional Grief Therapy significantly reduced maladaptive grief, PTSD, and depressive symptoms with large effect sizes (Hill et al., 2019).

In terms of the group format, many researchers have developed communal interventions to support individuals who have lost someone close to them—including family members—to foster a sense of community and support (e.g., O'Brien et al., 2022; Pelacho-Rios & Bernabe-Valero, 2023). Our findings support this notion, demonstrating that group interventions effectively reduced grief symptoms, improved emotional regulation, and, perhaps most critically, increased perceived social support (Linder et al., 2022; Siddaway et al., 2015). For instance, an 8-week school-based grief group treatment program led to significant reductions in grief symptoms and emotion dysregulation, along with an increase in social support among children and youth (Linder et al., 2022). Similarly, the child bereavement service showed a medium-sized decrease in psychological symptoms over time, with a significant number of participants exhibiting reliable and clinically significant improvement (Siddaway et al., 2015). These findings highlight the benefits of group interventions in providing social and emotional support to bereaved individuals. However, comparable social and emotional support data in individual and family interventions were not collected, limiting direct comparisons. As such, while these findings suggest benefits for group settings, further research is needed to assess whether other formats might offer similar advantages.

Finally, familial interventions have been used for decades to support grieving children, with the primary intention of involving surviving caregivers to ensure the child has present attachment figures in their lives (e.g., Black & Urbanowicz, 1987; Sandler et al., 2010)—a crucial factor for promoting long-term well-being (Field, 2006). Our findings indicate that family-based interventions were most effective for reducing maladaptive grief reactions and suicide ideation among relatives of suicide victims (de Groot et al., 2010), reducing problematic grief and social detachment/insecurity among parentally bereaved youth (Sandler et al., 2010), and improving prolonged grief disorder, PTSD, depression, and problem behaviours in both individual and familial contexts (Spuij et al., 2015). As research has demonstrated in the past (Bergman et al., 2017), the Family Bereavement Program (FBP) was particularly noteworthy for its long-term benefits, showing sustained reductions in problematic grief and improvements in social detachment/insecurity at six-year follow-up (Sandler et al., 2010). Similarly, family-based cognitive-behavioural grief therapy was effective in addressing maladaptive grief and reducing suicide ideation (de Groot et al., 2010). Ultimately, family-level interventions were effective in addressing maladaptive grief reactions and reducing psychological symptoms. While some studies focused on relatives of suicide victims (e.g., de Groot et al., 2010), other research (e.g., Sandler et al., 2010) supports the use of family-based approaches across diverse bereavement scenarios. These findings suggest that family interventions are broadly applicable beyond suicide-related loss, yet further controlled studies are needed to investigate the relative effectiveness of these interventions across different types of grief and circumstances.

In conclusion, when comparing the three intervention formats, individualized interventions appear most effective for addressing specific personal grief symptoms based on the outcomes observed in this review. However, due to the variability in study designs and outcome measures, direct comparisons across intervention types remain limited. These findings provide valuable insights into potentially beneficial approaches, but further research is needed to validate and compare these formats. Our results further suggest that this makes such interventions suitable for children and adolescents experiencing significant prolonged grief disorder, depression, PTSD, and concerning behaviours. These interventions are tailored to the individual’s needs, allowing for targeted therapeutic approaches.

Group interventions, on the other hand, excel in providing social and emotional support, fostering a sense of community among participants. They are particularly beneficial in settings where peer support and shared experiences can enhance coping mechanisms, as evidenced by significant improvements in emotional regulation and social support. Family-focused interventions offer a unique advantage in addressing grief within the context of family dynamics, especially for those affected by suicide and parental loss. These interventions ensure that both the child and their caregivers receive support, which can be crucial for long-term well-being. Given the variability in outcomes and the lack of comparable measures across formats, definitive conclusions about the superiority of any one intervention format cannot be drawn. The recommendations in this review are provisional, reflecting trends in available evidence, and other intervention formats may be equally effective under different circumstances.

### Limitations

This scoping review has several limitations. The small number of included studies, due to limited research in grief interventions for parentally bereaved children, may affect the generalizability of our findings. Cultural and contextual differences across studies conducted in various countries with differing norms and support systems for bereaved children may influence intervention effectiveness. e.g., Kalantari et al. (2012) found that trauma-informed writing interventions were particularly effective among war-bereaved adolescents in a refugee context. Further research should investigate how interventions can be tailored to different cultural needs. Also, the heterogeneity of participant characteristics, intervention content, and outcome measures across studies complicates direct comparisons and outcome synthesis.

Additionally, many studies relied on self-reported outcomes, which can introduce biases such as social desirability and recall, potentially affecting the accuracy of results. The lack of long-term follow-up in many studies also limits our understanding of the sustained impact of interventions. Variability in program duration and structure among the studies further complicates determining the optimal length and intensity for grief interventions. Moreover, while including multiple methodological designs (e.g., randomized controlled trials, quasi-experimental designs, and pre-post studies) can be considered a strength, it also introduces variability in the quality and rigour of reviewed findings.

Finally, the focus on interventions for parentally bereaved children excludes other forms of childhood grief (e.g., disenfranchised grief, ambiguous loss), limiting the applicability of findings to other bereaved populations. Future research should assess outcomes associated with various types of childhood grief to provide a more comprehensive understanding of intervention effectiveness.

### Practical Implications and Future Research

The findings from this scoping review underscore the importance of tailoring grief interventions to the specific needs of parentally bereaved children. Individual interventions should be prioritized for children experiencing significant prolonged grief, depression, PTSD, and problem behaviours, as they allow for targeted therapeutic approaches. Group interventions, which enhance social and emotional support through peer connections, are particularly beneficial in school settings or community programs. Familial interventions are crucial for addressing grief within the family context, providing support to both children and their caregivers, especially in cases of suicide and parental loss.

Future researchers should aim to increase the number of rigorous, long-term studies to understand these interventions’ sustained impacts better. It is also essential to explore the cultural and contextual factors that may influence the effectiveness of grief interventions, ensuring they are adaptable to diverse populations. Expanding the scope of research to include other forms of childhood bereavement, such as the loss of siblings or other significant loved ones, will work to provide a more comprehensive understanding of how to support all grieving children. Additionally, developing standardized guidelines for implementing and evaluating grief interventions, such as those proposed by Breen and colleagues (2017), will enhance the consistency and comparability of future studies. Of note, this research should be done with a specific emphasis on parentally bereaved children.

## Conclusion

This scoping review highlights the effectiveness of individual, group, and family interventions for parentally bereaved children. Individual interventions, particularly cognitive-behavioural techniques, effectively reduce prolonged grief disorder, depression, PTSD, and concerning behaviours. Group interventions provide social and emotional support, while family interventions are crucial for addressing grief within the family context, especially in cases of suicide and parental loss. Support providers (e.g., therapists, school counselors, social workers) can use these findings to guide intervention selection. For instance, individual interventions may be prioritized for children exhibiting severe PTSD or prolonged grief symptoms, while group interventions may be more suitable in school settings to foster peer support and emotional regulation. However, given the variability in study designs and outcomes, these recommendations should be considered provisional. Further research is needed to confirm the comparative effectiveness of different formats across diverse populations and settings.

Moreover, limitations such as small study numbers, variability in program structure, and reliance on self-reported outcomes persist. Future research should focus on long-term impacts, expand to other forms of childhood bereavement, and consider cultural factors. Standardizing guidelines will also enhance the consistency and effectiveness of grief interventions.

## Data Availability

The datasets generated during the current study are available from the corresponding author upon reasonable request.*

## References

[bibr1-00302228251334249] Abuhegazy H. M. Elkeshishi H. I. (2017). The effect of support group therapy on parentally bereaved adolescents: A randomized controlled trial. MOJ Addiction Medicine & Therapy, 3(5), 150–156. 10.15406/mojamt.2017.03.00055

[bibr2-00302228251334249] Alvis L. Zhang N. Sandler I. N. Kaplow J. B. (2023). Developmental manifestations of grief in children and adolescents: Caregivers as key grief facilitators. Journal of Child & Adolescent Trauma, 16(2), 447–457. 10.1007/s40653-021-00435-0 PMC8794619 35106114

[bibr3-00302228251334249] Angelhoff C. Sveen J. Alvariza A. Weber-Falk M. Kreicbergs U. (2021). Communication, self-esteem and prolonged grief in parent-adolescent dyads, 1–4 years following the death of a parent to cancer. European Journal of Oncology Nursing: The Official Journal of European Oncology Nursing Society, 50, Article 101883. 10.1016/j.ejon.2020.101883 33360292

[bibr59-00302228251334249] Arksey H. O’Malley L. (2005). International Journal of Social Research Methodology, 8(1), 19–32. 10.1080/1364557032000119616

[bibr4-00302228251334249] Bergman A. S. Axberg U. Hanson E. (2017). When a parent dies–a systematic review of the effects of support programs for parentally bereaved children and their caregivers. BMC Palliative Care, 16, 1–15. 10.1186/s12904-017-0223-y PMC5553589 28797262

[bibr5-00302228251334249] Black D. Urbanowicz M. A. (1987). Family intervention with bereaved children. The Journal of Child Psychology and Psychiatry and Allied Disciplines, 28(3), 467–476. 10.1111/j.1469-7610.1987.tb01767.x 3597568

[bibr6-00302228251334249] Boelen P. A. Lenferink L. I. Spuij M. (2021). CBT for prolonged grief in children and adolescents: A randomized clinical trial. American Journal of Psychiatry, 178(4), 294–304. 10.1176/appi.ajp.2020.2005054 33472391

[bibr7-00302228251334249] Breen L. J. Hall C. W. Bryant R. A. (2017). A clinician's quick guide of evidence‐based approaches: Prolonged grief disorder. Clinical Psychologist, 21(2), 153–154. 10.1111/cp.12124

[bibr8-00302228251334249] Breier A. Kelsoe J. R. Kirwin P. D. Beller S. A. Wolkowitz O. M. Pickar D. (1988). Early parental loss and development of adult psychopathology. Archives of General Psychiatry, 45(11), 987–993. 10.1001/archpsyc.1988.01800350021003 2972265

[bibr9-00302228251334249] Burns M. Griese B. King S. Talmi A. (2020). Childhood bereavement: Understanding prevalence and related adversity in the United States. American Journal of Orthopsychiatry, 90(4), 391–405. 10.1037/ort0000442 31999137

[bibr10-00302228251334249] Chater A. M. Howlett N. Shorter G. W. Zakrzewski-Fruer J. K. Williams J. (2022). Reflections on experiencing parental bereavement as a young person: A retrospective qualitative study. International Journal of Environmental Research and Public Health, 19(4), 2083. 10.3390/ijerph19042083 PMC8872611 35206275

[bibr11-00302228251334249] Christ G. H. Raveis V. H. Siegel K. Karas D. Christ A. E. (2005). Evaluation of a preventive intervention for bereaved children. Journal of Social Work in End-of-Life and Palliative Care, 1(3), 57–81. 10.1300/J457v01n03_05 17387069

[bibr12-00302228251334249] Cohen J. A. Mannarino A. P. Deblinger E. (2016). Treating trauma and traumatic grief in children and adolescents. Guilford Publications.

[bibr13-00302228251334249] Currier J. M. Holland J. M. Neimeyer R. A. (2007). The effectiveness of bereavement interventions with children: A meta-analytic review of controlled outcome research. Journal of Clinical Child and Adolescent Psychology: The Official Journal for the Society of Clinical Child and Adolescent Psychology, American Psychological Association, Division 53, 36(2), 253–259. 10.1080/15374410701279669 17484697

[bibr14-00302228251334249] De Groot M. Neeleman J. Van der Meer K. Burger H. (2010). The effectiveness of family-based cognitive-behaviour grief therapy to prevent complicated grief in relatives of suicide victims: The mediating role of suicide ideation. Suicide and Life-Threatening Behavior, 40(5), 425–437. 10.1521/suli.2010.40.5.425 21034206

[bibr15-00302228251334249] Dopp A. R. Cain A. C. (2012). The role of peer relationships in parental bereavement during childhood and adolescence. Death Studies, 36(1), 41–60. 10.1080/07481187.2011.573175 24567994

[bibr16-00302228251334249] Feigelman W. Cerel J. McIntosh J. L. Brent D. Gutin N. (2018). Suicide exposures and bereavement among American adults: Evidence from the 2016 general social survey. Journal of Affective Disorders, 227, 1–6. 10.1016/j.jad.2017.09.056 29045914

[bibr17-00302228251334249] Field N. P. (2006). Unresolved grief and continuing bonds: An attachment perspective. Death Studies, 30(8), 739–756. 10.1080/07481180600850518 16972370

[bibr18-00302228251334249] Fluegeman J. E. Schrauben A. R. Cleghorn S. M. (2013). Bereavement support for children: Effectiveness of Camp Erin from an occupational therapy perspective. Bereavement Care, 32(2), 74–81. 10.1080/02682621.2013.812820

[bibr19-00302228251334249] Giang C. Alvis L. Oosterhoff B. Kaplow J. B. (2024). Protective factors in the context of childhood bereavement: Youth gratitude, future orientation, and purpose in life. Omega, 0(0), Article 302228241246919. 10.1177/00302228241246919 38621174

[bibr20-00302228251334249] Guldin M. Li J. Pedersen H. S. Obel C. Agerbo E. Gissler M. Cnattingius S. Olsen J. Vestergaard M. (2015). Incidence of suicide among persons who had a parent who died during their childhood: A population-based cohort study. JAMA Psychiatry, 72(12), 1227–1234. 10.1001/jamapsychiatry.2015.2094 26558351

[bibr21-00302228251334249] Haine R. A. Ayers T. S. Sandler I. N. Wolchik S. A. (2008). Evidence-based practices for parentally bereaved children and their families. Professional Psychology: Research and Practice, 39(2), 113–121. 10.1037/0735-7028.39.2.113 PMC2888143 20585468

[bibr22-00302228251334249] Hamdan S. Berkman N. Lavi N. Levy S. Brent D. (2019). The effect of sudden death bereavement on the risk for suicide. Crisis. 10.1027/0227-5910/a000635 31859558

[bibr23-00302228251334249] Hill R. M. Oosterhoff B. Layne C. M. Rooney E. Yudovich S. Pynoos R. S. Kaplow J. B. (2019). Multidimensional grief therapy: Pilot open trial of a novel intervention for bereaved children and adolescents. Journal of Child and Family Studies, 28(11), 3062–3074. 10.1007/s10826-019-01481-x

[bibr24-00302228251334249] Howell K. H. Shapiro D. N. Layne C. M. Kaplow J. B. (2015). Individual and psychosocial mechanisms of adaptive functioning in parentally bereaved children. Death Studies, 39(5), 296–306. 10.1080/07481187.2014.951497 25848701

[bibr25-00302228251334249] Hung N. C. Rabin L. A. (2009). Comprehending childhood bereavement by parental suicide: A critical review of research on outcomes, grief processes, and interventions. Death Studies, 33(9), 781–814. 10.1080/07481180903142357 19798803

[bibr26-00302228251334249] Huss S. N. Ritchie M. (1999). Effectiveness of a group for parentally bereaved children. Journal for Specialists in Group Work, 24(2), 186–196. 10.1080/01933929908411429

[bibr27-00302228251334249] Johnson J. G. Cohen P. Kasen S. Brook J. S. (2002). Childhood adversities associated with risk for eating disorders or weight problems during adolescence or early adulthood. American Journal of Psychiatry, 159(3), 394–400. 10.1176/appi.ajp.159.3.394 11870002

[bibr28-00302228251334249] Kailaheimo-Lönnqvist S. Kotimäki S. (2020). Cause of parental death and child's health and education: The role of parental resources. SSM - population health, 11, Article 100632. 10.1016/j.ssmph.2020.100632 PMC7426579 32817879

[bibr29-00302228251334249] Kalantari M. Yule W. Dyregrov A. Neshatdoost H. Ahmadi S. J. (2012). Efficacy of writing for recovery on traumatic grief symptoms of afghani refugee bereaved adolescents: A randomized control trial. Omega, 65(2), 139–150. 10.2190/OM.65.2.d 22953510

[bibr30-00302228251334249] Kentor R. A. Kaplow J. B. (2020). Using superheroes in grief counseling with children and adolescents. In Superhero grief (pp. 214–220). Routledge.

[bibr31-00302228251334249] Kwok O. M. Haine R. A. Sandler I. N. Ayers T. S. Wolchik S. A. Tein J. Y. (2005). Positive parenting as a mediator of the relations between parental psychological distress and mental health problems of parentally bereaved children. Journal of Clinical Child and Adolescent Psychology: The Official Journal for the Society of Clinical Child and Adolescent Psychology, American Psychological Association, Division 53, 34(2), 260–271. 10.1207/s15374424jccp3402_5 15901226

[bibr32-00302228251334249] Linder L. Lunardini M. Zimmerman H. (2022). Supporting childhood bereavement through school-based grief group. Omega, 89(2), 741–758. 10.1177/00302228221082756 35357962

[bibr33-00302228251334249] Liu C. Grotta A. Hiyoshi A. Berg L. Rostila M. (2022). School outcomes among children following death of a parent. JAMA Network Open, 5(4), e223842. 10.1001/jamanetworkopen.2022.3842 PMC8994122 35394516

[bibr34-00302228251334249] Lutzke J. R. Ayers T. S. Sandler I. N. Barr A. (1997). Risks and interventions for the parentally bereaved child. In Wolchik S. A. Sandler I. N. (Eds.), Handbook of children's coping: Linking theory and intervention (pp. 215–243). Plenum Press. 10.1007/978-1-4757-2677-0_8

[bibr35-00302228251334249] MacKinnon C. J. Smith N. G. Henry J. Milman E. Cohen S. R. (2015). Meaning-based group counseling for bereaved youth: Results of a pilot randomized controlled trial. Death Studies, 39(8), 512–523. 10.1080/07481187.2015.1068242

[bibr36-00302228251334249] McClatchey I. S. Wimmer J. S. (2014). Coping with parental death as seen from the perspective of children who attended a grief camp. Qualitative Social Work, 13(2), 221–236. 10.1177/1473325012465104

[bibr37-00302228251334249] Melhem N. M. Porta G. Shamseddeen W. Payne M. W. Brent D. A. (2011). Grief in children and adolescents bereaved by sudden parental death. Archives of General Psychiatry, 68(9), 911–919. 10.1001/archgenpsychiatry.2011.101 PMC4017669 21893658

[bibr38-00302228251334249] Metel M. Barnes J. (2011). Peer‐group support for bereaved children: A qualitative interview study. Child and Adolescent Mental Health, 16(4), 201–207. 10.1111/j.1475-3588.2011.00601.x 32847193

[bibr39-00302228251334249] Moor N. de Graaf P. M. (2016). Temporary and long-term consequences of bereavement on happiness. Journal of Happiness Studies, 17(3), 913–936. 10.1007/s10902-015-9624-x

[bibr60-00302228251334249] Munn Z. Peters M. Stern C. Tufanaru C. McArthur A. Aromataris E. (2018). Systematic review or scoping review? Guidance for authors when choosing between a systematic or scoping review approach. BMC Medical Research Methodology, 18. 10.1186/s12874-018-0611-x PMC6245623 30453902

[bibr40-00302228251334249] Neimeyer R. A. Currier J. M. (2009). Grief therapy: Evidence of efficacy and emerging directions. Current Directions in Psychological Science, 18(6), 352–356. 10.1111/j.1467-8721.2009.01666.x

[bibr41-00302228251334249] O'Brien K. M. Cannon G. Stearns A. K. Walsh C. Hill E. M. (2022). I'm not alone: A group intervention for grieving children. Journal of Prevention and Health Promotion, 3(3), 395–414. 10.1177/26320770221098730

[bibr42-00302228251334249] Pelacho-Rios L. Bernabe-Valero G. (2023). The loss of a child, bereavement and the search for meaning: A systematic review of the most recent parental interventions. Current Psychology, 42(29), 25931–25956. 10.1007/s12144-022-03703-w PMC9452864 36097578

[bibr44-00302228251334249] Pynoos R. S. Nader K. (1990). Children's exposure to violence and traumatic death. Psychiatric Annals, 20(6), 334–344. 10.3928/0048-5713-19900601-05

[bibr45-00302228251334249] Rosner R. Kruse J. Hagl M. (2010). A meta-analysis of interventions for bereaved children and adolescents. Death Studies, 34(2), 99–136. 10.1080/07481180903492422 24479177

[bibr46-00302228251334249] Rosner R. Pfoh G. Kotoučová M. (2011). Treatment of complicated grief. European Journal of Psychotraumatology, 2(1), 7995. 10.3402/ejpt.v2i0.7995 PMC3402114 22893810

[bibr47-00302228251334249] Ross D. Hayes B. (2004). Intervening with groups of bereaved pupils. Educational and Child Psychology, 21(3), 95. 10.3402/ejpt.v2i0.7995

[bibr49-00302228251334249] Sandler I. N. Ayers T. S. Wolchik S. A. Tein J.-Y. Kwok O.-M. Haine R. A. Twohey-Jacobs J. Suter J. Lin K. Padgett-Jones S. Weyer J. L. Cole E. Kriege G. Griffin W. A. (2003). The Family Bereavement Program: Efficacy evaluation of a theory-based prevention program for parentally bereaved children and adolescents. Journal of Consulting and Clinical Psychology, 71(3), 587–600. 10.1037/0022-006X.71.3.587 12795581

[bibr50-00302228251334249] Sandler I. N. Ma Y. Tein J. Y. Ayers T. S. Wolchik S. Kennedy C. Millsap R. (2010). Long-term effects of the family bereavement program on multiple indicators of grief in parentally bereaved children and adolescents. Journal of Consulting and Clinical Psychology, 78(2), 131–143. 10.1037/a0018393 PMC2874830 20350025

[bibr51-00302228251334249] Shapiro D. N. Howell K. H. Kaplow J. B. (2014). Associations among mother–child communication quality, childhood maladaptive grief, and depressive symptoms. Death Studies, 38(3), 172–178. 10.1080/07481187.2012.738771 24524545

[bibr52-00302228251334249] Siddaway A. P. Wood A. M. Schulz J. Trickey D. (2015). Evaluation of the CHUMS child bereavement group: A pilot study examining statistical and clinical change. Death Studies, 39(2), 99–110. 10.1080/07481187.2014.913085 25153045

[bibr53-00302228251334249] Simon N. M. (2013). Treating complicated grief. JAMA, 310(4), 416–423. 10.1001/jama.2013.8614 PMC4530627 23917292

[bibr54-00302228251334249] Slomine B. S. Copeland-Linder N. (2021). Early-life adversity and executive functioning—highlighting an urgent need for identification, prevention, and intervention in childhood. JAMA Pediatrics, 175(11), e212517. 10.1001/jamapediatrics.2021.2517 34309640

[bibr55-00302228251334249] Spuij M. Dekovic M. Boelen P. A. (2015). An open trial of 'grief‐help': A cognitive–behavioural treatment for prolonged grief in children and adolescents. Clinical Psychology & Psychotherapy, 22(2), 185–192. 10.1002/cpp.1877 24227661

[bibr56-00302228251334249] Thompson A. Gardner M. Russell C. Steinberg D. (2019). Fact sheet: Grief in children and adolescents. Society of Pediatric Psychology. https://pedpsych.org/fact_sheets/grief-children-and-adolescents/

[bibr57-00302228251334249] Tillman K. S. Prazak M. (2018). Kids supporting kids: A 10‐week small group curriculum for grief and loss in schools. Counselling and Psychotherapy Research, 18(4), 395–401. 10.1002/capr.12190

[bibr58-00302228251334249] Tricco A. C. Lillie E. Zarin W. O'Brien K. K. Colquhoun H. Levac D. Moher D. (2018). PRISMA extension for scoping reviews (PRISMA-ScR): Checklist and explanation. PLOS Med, 15(11), Article e1000297. 10.1371/journal.pmed.1000297 30178033

